# An Account of a Disease Which, until Lately, Proved Fatal to a Great Number of Infants in the Lying-In Hospital of Dublin; with Observations on Its Causes and Prevention

**Published:** 1792

**Authors:** Joseph Clarke

**Affiliations:** Master of the Hospital above Mentioned, and M. R. I. A.


XI. *An Account of a Diſeaſe which, until lately, proved fatal to a great Number of Infants in the Lying-in Hoſpital of Dublin; with Obſervations on its Cauſes and Prevention.* By Joſeph Clarke, *M. D. Maſter of the Hoſpital above mentioned, and M. R. I. A. — From the Tranſactions of the Royal Iriſh Academy,* 1789. 4to. Dublin, 1789.

LYING-IN Hoſpitals are inſtitutions of ſuch recent date, and ſo few in number, that hitherto we may conſider them as in a ſtate of infancy. Excepting ſome portion of the Hotel Dieu of Paris, which has been long allotted lotted to the relief of poor pregnant women, I know of none that have exifted above forty years, and very few can lay claim even to this antiquity. It can hardly appear unreafonable, therefore, to fuppofe that imperfections ftill exift in their management, which time and accurate comparifon may ferve to detect: and although fuch eftablifhments be at prefent confined to a few of the capital cities in Europe, it is probable their number will increafe as their good effects in fociety are experienced. It is hoped, therefore, that a few facts and obfervations, tending to point out a confiderable fource of error in an extenfive lying-in hofpital, may be deemed worthy of public notice; both prefent and future inftitutions of this nature may, perhaps, derive fome ufeful information from fuch enquiry.

Several years ago, in attempting to afcertain the nature of the difeafe which is the fubject of the following remarks, I found the doctrines contained in moft medical books of very little ufe: all the morbid caufes, commonly fuppofed to produce difeafes in infancy, appeared to me inadequate to an explanation of its phænomena. Doubts of courfe arofe in my mind, fome of which have been already ftated to the public.

public*. At length I was tempted to hazard a conjecture, which then appeared probable, and which succeeding events seem to have confirmed. A sketch of the evidence is here, with deference, submitted to the candid consideration of physicians, and of this Academy.

At the conclusion of the year 1782, of seventeen thousand six hundred and fifty infants born alive in the Lying-in Hospital of this city, two thousand nine hundred and forty-four had died within the first fortnight †, that is nearly every sixth child, or about seventeen in the hundred. This was obviously a large proportion of deaths, as we shall prove more particularly hereafter. The disease which carried off most of these children, perhaps nineteen of twenty, was general convulsions, or what our nurse-tenders have been long in the habit of calling the nine-day fits, as constantly occurring within the first nine days after birth. As this disease has hitherto yielded to no remedy, I have been always more engaged in attending to its prevention than cure. I am chiefly indebted for its history, therefore, to the united

* See Observations on the Properties commonly attributed by medical Writers to human Milk, &c. Transactions of the Royal Irish Academy, Vol. II. and London Medical Journal, Vol. XI.

† See abstract of registry at the end of this essay.

reports reports of ſeveral of our moſt experienced nurſe-tenders. I took down their remarks ſeparately, and from the whole collected what follows.

In general it has been obſerved that ſuch children as are diſpoſed to whine and cry much from their birth, and ſuch as are ſubject to heavy deep ſleeps, or ſtartings in their ſleep, are peculiarly apt to fall into convulſive affections. Twiſting of the upper extremities, while awake, without any evident cauſe; a livid circle about the lips, and ſudden changes of colour in the countenance, have now and then been thought to portend the nine-day fits Screwing and gathering of the mouth into a purſe, accompanied at intervals with a particular kind of ſhrieking, well known to the experienced nurſe-tenders, are reckoned ſure, and by no means diſtant, forerunners. Sometimes previous to theſe ſymptoms, and ſometimes along with them, the infants are obſerved to be unuſually greedy for ſucking at the breaſt, or feeding by the ſpoon; laxatives given, in ſuch ſituations, ſeldom fail to operate freely, ſometimes bringing away greeniſh, ſlimy, or knotty ſtools; though not unfrequently they are of a natural yellow colour, as I myſelf have more than once ſeen.

Generally with one or more of theſe ſymptoms preceding, but ſometimes without any warning whatever, the infants are ſeized with violent irregular contractions and relaxations of the muſcular frame, but particularly of thoſe of the extremities and face. Theſe convulſive motions recur at uncertain intervals, and produce various effects. In ſome the agitation is very great; the mouth foams; the thumbs are riveted into the palms of the hands; the jaws are locked from the commencement, ſo as to prevent the actions of ſucking and ſwallowing; and any attempts to wet the mouth or fauces, or to adminiſter medicines, ſeem to aggravate the ſpaſms very much; the face becomes turgid, and of a livid hue, as do moſt other parts of the body. From this circumſtance, and from the ſhorter duration of the diſeaſe, when it occurs in this form, the nurſes reckon this a different ſpecies, and call it the black fits. The conflict in ſuch caſes laſts from about eight to thirty hours, and in ſome very rare caſes to about forty hours, when the powers of nature ſink exhauſted and overpowered, as it were, with their own exertions.

It much more frequently happens, however, that the ſpaſmodic contractions are not ſo ſtrong as above deſcribed; that the extremities are rather ther twifted than convulfed; that the power of fucking, but more certainly of deglutition, is not loft till near death; that the mouth foams lefs; and that the paroxyfms recurring at more diftant intervals, continue to harafs the patient from three to five days, and in fome rare inftances to feven and even nine. During all this period the face remains pale; and the body, from being perhaps very plump, is reduced to a moft miferable fpectre by emaciation and difeafe. This the nurfes confider as a fecond fpecies, and call it the white fits.

Both thefe fuppofed fpecies, which may perhaps be more juftly confidered as varieties of the fame difeafe, agree in conftantly attacking within nine days from birth, and moft frequently about the falling off of the umbilical chord. This is an event which generally takes place from the fourth to the fixth or feventh day. Diarrhœa is a conftant concomitant of both fpecies. Long and fad experience have found them alfo to be both equally fatal, infomuch, that the memory of the oldeft perfon does not furnifh an inftance of one being cured.

In order to place my ideas of the caufe of this fatal difeafe in the cleareft point of view, I find it neceffary to have recourfe to extracts from a letter written by me in the year 1783 to the late Doctor Hutchefon, who was then confulting phyfician to the hofpital in queftion.

In this letter, which was written after having feen fome of the beft regulated Lying-in Hofpitals in London, I ftated to Doctor Hutchefon,

That in an old hofpital, which preceded the prefent, but inftituted by and under the care of the fame gentleman, and in a lefs airy part of Dublin, of three thoufand feven hundred and forty-fix children therein born, only two hundred and forty-one died within the firft month*, which are in the proportion of one to fifteen and a half, or from fix to feven in the hundred.

That during a period of five or fix years, in the Britifh Lying-in Hofpital, London, of three thoufand fix hundred and eleven therein born, only one hundred and forty-fix died, within the firft three weeks or month, which are as one to twenty-five, or four in the hundred.

That in the London Lying-in Hofpital I was pofitively affured that the death of an infant was

* See the cafe of Mr. Moffe, offered to the confideration of the Irifh Houfe of Commons in the year 1755.

a rare a rare occurrence. It is there computed with ſome confidence (for I was told that no written account is kept) that the number of ſtill-born infants far exceeds the number of thoſe dying after birth. The proportion of ſtill-born we know to be about a twentieth part, or five in the hundred.

That near forty years ago, when the diſeaſes of children were leſs underſtood, and more eſpecially the ſalutary practice of inoculation, Doctor Short computed from ſome very extenſive regiſters, that London loſt thirty-nine per cent. under the age of two years—Edinburgh and Northampton thirty-four or thirty-five—Sheffield twenty-eight—country places from twenty to twenty-eight;—whereas in the Dublin hoſpital there was loſt a number equal to half of that loſt in many of theſe places, and nearly equal to the whole of that in ſome of them, in two weeks, or in about the fiftieth part of the ſame ſpace of time. From which, and ſome other conſiderations of leſs weight, I thought the uncommon mortality of children in the Dublin Lying-in Hoſpital ſatisfactorily proved.

I then ventured to hazard ſome conjectures concerning the cauſes of a mortality, by which ſo many uſeful lives were loſt to the ſtate.

1ſt, Foul air, or an impure atmoſphere;

2d, Neglect of keeping the children clean and dry;

3d, Irregularity in the manner of living of their mothers, more eſpecially in the abuſe of ſpirituous liquors,—were the cauſes which appeared to me the moſt probable, either ſeparately or perhaps combined; but I ſuſpected that the firſt, viz. an impure or phlogiſticated atmoſphere, contributed moſt powerfully to the general calamity. For,

Firſt. I remarked to him that public regiſters proved the mortality of children to increaſe proportionably with the ſize of towns; and that the larger towns are, the more numerous are the cauſes which have a tendency to taint their atmoſphere, and thereby render it leſs fit for the purpoſes of ſalutary reſpiration.

Secondly. That in private practice phyſicians in the city of Dublin did not find the mortality of infants in any degree ſo conſiderable as our regiſtry proved it to be in the Hoſpital, a proof that there was here ſome peculiar exciting cauſe of diſeaſe.

Thirdly. That the difference between the mortality of the children in the old hoſpital and in the preſent one, when under the management of of the ſame eminent character, Mr. Moſſe, afforded the ſtrongeſt evidence in favour of this conjecture. Such difference could not be ſuppoſed to ariſe from any different method of feeding or cloathing them, or in the exhibition of medicines; to me it ſeemed to originate from a difference in the apartments and accommodations of the women. In the former, which was an old houſe, and never deſigned for an hoſpital, were one or two, or at moſt three beds in a room, to each of which there muſt have been a door, and one or two, perhaps three windows; whereas in the latter were eight beds in the ſame room, and only one door properly ſpeaking *, with three windows in ſome, and two in others; whence it is evident that the ſupply of freſh air in each being nearly on an equality, it muſt be much ſooner corrupted by the reſpiration, lochial diſcharges, and other effluvia of eight

* There is indeed a ſecond door to each of our large wards; but as it opens into a ſmall ward, containing two beds, it is probable the air derived from ſuch communication is not very ſalubrious. The dimenſions of our large wards, in the front of the hoſpital, are 36 feet by 23, and 13 in height: in the rear 33½ by 23, and of equal height. The ſmall wards in front are 19 by 12½; and in rear, 18 by 13½.

women and as many children, than by thoſe of two or three.

Fourthly. I obſerved, in farther confirmation of this doctrine, that the Britiſh Lying-in Hoſpital in London, which is very favourable to the lives of infants, was an old building, which ſeemed not to have been originally deſigned for an hoſpital; in it there were but ſix beds in a room with one door, one ſmall and three large windows, with a ventilator to each of the latter; that their beds had curtains, but no canopies as in Dublin, and that the utmoſt cleanlineſs was in every reſpect obſerved. That in the London Lying-in Hoſpital, which is an elegant modern building, there are but ſeven beds to a ward, with two large and four ſmall windows to each, one door with a large ventilator over it, the ceilings lofty and perforated by an air-pipe of ſeveral inches diameter, which paſſes out at ſome part of the roof. Here alſo the moſt ſcrupulous cleanlineſs is obſerved, and large ſupplies of clean linen given both for beds, women and infants; and here the death of an infant is a rare occurrence.

Laſtly. I alledged it was by no means inconſiſtent with analogy or reaſon to ſuppoſe that the accumulated effluvia ariſing from the bodies of puerperal puerperal women and children in lying-in hofpitals might acquire qualities peculiarly noxious to the delicate frame of infants. That in other hofpitals and gaols, as the pernicious effects of accumulated human effluvia have been often experienced by robuft adults, it is poffible that degrees of contagion inferior to thefe may prove fatal to infants. I concluded with quoting the authority of Arbuthnot, who has obferved "that "the air of cities is very unfriendly to infants "and children; for that as every animal is adap- "ted by nature to the ufe of frefh and free air, "the tolerance of air replete with fulphureous "fteams of fuel and the perfpirable matter of "animals (as that of cities) is the effect of "habit which young creatures have not yet ex- "perienced*;" and that if the air of cities be unfriendly, *a fortiori*, fo muft the air of hofpitals in cities, and that in proportion to their want of ventilation.

To thefe reafons I might have added, on the authority of Doctor Prieftley, that healthy animals almoft always die of convulfions on being put into air in which other animals have died, after breathing it as long as they could; and that moft other kinds of air, noxious to animal

* Effay concerning the Effects of Air on human Bodies.

life, life, produce fimilar effects. See Experiments and Obfervations on different Kinds of Air, Vol. I. page 71.

Viewing the fubject in this light, I propofed a number of alterations intended for the more complete ventilation of the hofpital, and for which I was principally indebted to Mr. White's excellent work on the management of lying-in women. My obfervations had the effect I wifhed with Doctor Hutchefon and the medical governors. Apertures of a confiderable fize were made in the ceilings of each ward, which have been fince changed for air pipes of fix inches diameter. Three holes, of an inch diameter, were bored, in an oblique direction, through each window frame at top. The upper parts of the doors, opening into the gallery, were alfo perforated with a great number of holes. By thefe means a free and eafy paffage was given to the air through the wards at all times, and executed in fuch a manner as to put it out of the power of nurfe-tenders or patients to control it. Since the above period alfo the number of beds in the large wards have been reduced to feven, and feveral changes made in their conftruction, which render them more airy, and more eafily kept clean. The confequences have been favourable far beyond the expectation of every perfon concerned. The nine-day fits are become vifibly lefs frequent; and the abftract of our regiftry fhews the fact at firft view to the moft inattentive obferver. Of eight thoufand and thirty-three children born fince the above period, only four hundred and nineteen have died in the hofpital; that is nearly one in nineteen and a third, or from five to fix in the hundred. Had the mortality of infants been in this proportion fince the commencement of the Dublin hofpital, the number of children dead would have been fomewhat about thirteen hundred, inftead of the prefent number, three thoufand three hundred and fixty-three; or in other words, above two thoufand lives would have been faved to the community.

That this diminution of mortality is to be attributed to improvements in ventilation can admit, I think, of little doubt. No other new mode of management has been of late practifed to account for it. No other remedies ufed than fuch as have been tried a thoufand times unfuccefsfully. I know it has been objected, that it may be owing to their mothers now remaining a fhorter fpace of time in the hofpital than formerly. In order to afcertain whether this this be a matter of fact, I have, for the laſt two years, had an entry made of the day on which each infant died; the number dead has been one hundred and fourteen, and they have died on the following days after their birth:

| 12th day, | 11th, | 10th, | 9th, | 8th, | 7th, | 6th, | 5th, | 4th, | 3d, | 2d, | 1ſt. | Total. |
|---|---|---|---|---|---|---|---|---|---|---|---|---|
| 1 died. | 0. | 3. | 3. | 5. | 24. | 37. | 18. | 6. | 5. | 10. | 2. | 114 died. |

Hence it is obvious that the fatal days are the fifth, the ſeventh, but eſpecially the ſixth; and either of theſe are undoubtedly much within the average day of the diſcharge of our patients. Beſides, the early diſcharge of patients did not commence in any one year, as the leſſened mortality of infants did; it aroſe from gradual increaſe in the number of poor demanding admiſſion; and I am happy to add, that ſome late very liberal donations, and a conſequent increaſe in the number of our beds, have put an end to the neceſſity of this diſagreeable expedient, adopted ſolely with a view of affording indiſcriminate relief.

It might naturally be ſuppoſed that an atmoſphere, which we have endeavoured to prove injurious to the health of infants, would alſo ſomewhat affect the chances of life in their mothers. The fact, however, certainly is, that on an average fewer women have died in childbed bed in the Dublin hofpital than in moft other lying-in hofpitals, (Compare the abftract at the end of this effay with facts contained in the poft-fcript to Mr. White's treatife on the management of pregnant and puerperal women.) Here then a queftion arifes, why fhould infants be fo much more liable to injury from an impure atmofphere than adults? Is it poffible that mothers fhall efcape with impunity and their children perifh? This, I own, puzzled me extremely, and had almoft made me doubt of what I confidered a fact, fupported by the ftrongeft probable evidence. By accident, however, in looking over a differtation on the food and difcharges of the human body, by our celebrated countryman, the late Doctor Bryan Robinfon, I found fome facts and obfervations which appear to me to go a great way towards an explanation.

In order to make thefe facts intelligible to perfons not very converfant in fuch fpeculations, I muft premife, that Doctor Prieftley has fully proved one great and indifpenfable ufe of refpiration to be to carry off or leffen a certain quality in the blood, which is known by the name of phlogifton. That this can only be done by pure air. That by the addition of phlogifton to blood it acquires a deep black colour; and by its avolation, that blood returns to its natural florid hue.

Now Doctor Robinfon found by experiment*, that the weight of the heart, in refpect to the weight of the body, is greater in children than in grown bodies, and that their quantity of blood is proportional to the weight of the heart. He found alfo, that the quantity of blood, which flows through the lungs in a given time, in proportion to the mafs of circulating fluids, is greater in children than in grown bodies; and that this proportion leffens continually from the birth till bodies arrive at their growth. Hence he remarks, that as the blood of children paffes oftener through the lungs, it is more fluid and of a brighter colour than the blood of grown perfons.

If this be a true picture of the conftitution of infants, we muft prefume that fuch peculiarities are intended to anfwer fome very important purpofes in the œconomy of young animals; and that in proportion as the intention of Nature is in thefe refpects fruftrated, the effects will be more or lefs feverely felt. Would it be deem-

* See page 13, et feq. of his work.

ed a conjecture, exceeding the bounds of probability, to suspect that the avolation of a very large quantity of phlogiston, and its due separation from the mass of blood by pure air, may be essentially necessary to the growth of young animals; and that this may be one reason why the impure air of cities has, in all ages, been particularly destructive to their health?

With a view of reducing the nine day fits to its proper genus and species in nosology, I have turned over the works of some of our best writers on this subject. The only genus to which I think it can with any propriety be reduced, is that of *eclampsia* or *convulsion des enfans* of Sauvages. But although under this generic title he describes seventeen species, there is not one of them to which it bears an exact resemblance. The *eclampsia neophytorum* of Vander Monde is widely different, as any one may easily see by casting an eye over the history of both. As most of the species enumerated by Sauvages are symptomatic, and as he distinguishes several of them from various kinds of deleterious substances taken into the system; as *eclampsia ab atropa*, *cicuta*, &c. perhaps we might with equal propriety add *eclampsia ab atmosphæra phlogisticata*.

There

There is a ſporadic diſeaſe in Minorca and ſome other countries ſo very like the nine day fits, in ſome particulars, that it may be worth while here to collect, under one point of view, a few extracts concerning it. Noſologiſts have given it the title of triſmus naſcentium. "In hac urbe "affli&ctantur plurimi infantes, adeo feroci con-"vulſione mandibulæ inferioris, ut ea appre-"henſi, nullo poſſint motu illam movere, et "abhinc ſuctus lactis impeditur omnino.....Tot "interficit mala iſta convulſio, ac variolæ aut "morbilli.....In hoc periculum incurrunt recen-"ter nati uſque ad *nonum* ſuæ nativitatis diem, "eoque tranſacto, omne diſcrimen ceſſare do-"cuit ſemper experientia." For theſe and ſome other obſervations, from the writings of a Spaniſh phyſician, we are indebted to my friend Doctor Cleghorn's valuable treatiſe on the diſeaſes of Minorca. After the hiſtory of the diſeaſe, the doctor obſerves that is is needleſs to add the remedies preſcribed by the Spaniſh author, as he ingenuouſly confeſſes the diſeaſe to be ſo ſeldom curable, that in twenty years practiſe he had ſcarce known ſix to recover.

In Germany, Heiſter, de maxillæ ſpaſmo, obſerves, "Quod ſi ſponte, ſive e cauſa interna, "hic maxillæ ſpaſmus in infantibus, ut ſæpe

"vidi,

"vidi, contingit ut plurimum moriuntur et

"vix ullum ſervatum vidi; licet laudatiſſima

"remedia nervina et antiſpaſmodica internè

"atque externè quam ſolertiſſimè adhibita fue-

"rint."

Hofer, in the firſt volume of the Acta Helvetica, has given a long account of a diſeaſe not unfrequent in ſome parts of Switzerland, which Sauvages and Cullen ſeem to think of the ſame ſpecies with the preceding, but which differs from them very materially in ſome reſpects.—The title of his paper is, De tetano maxillæ inferioris in Infantibus. "Subjectum iſti ob-

"noxium," ſays he, "eſt infans, qui inter

"tertiam et *duodecimam* ætatis diem verſa-

"tur. Cura hujus morbi, quamvis valde

"lenta ſit, attamen ſi infans quintam a mor-

"bi invaſione diem tranſegerit certiſſime fe-

"lix eſt, ideoque dummodo tempus terere

"poſſumus, res in ſalvo poſita eſt." After giving an account of his method of cure, which conſiſts of a farrago of diſtilled waters, ſyrups and inert powders, as may be ſeen in Sauvages, he concludes, "hæc eſt methodus applicando-

"rum medicamentorum, quâ ex tribus ægro-

" tulis curæ meæ commiſſis plerumque unus

" gratiâ divinâ evaſit."

A late French author, Monſ. Fourcroy, in a treatiſe entitled Les Enfans elevés dans l'ordre de la Nature, remarks " Quand je ſuis arrivé

" en 1744 a St. Domingue, on ne pouvoit ele-

" ver des negrillons dans la plaine du Cap Fran-

" cois. Ils mouroient preſque tous, c'eſt a

" dire environ quatre vingt ſur cent, d'une ma-

" ladie appellée dans le pays *mal de machoire*

" ou *tetanos*, qui les emportoit dans les *neuf*

" premiers jours de leur naiſſance." This diſorder he informs us, when come on, is beyond the power of medicine, but that much may be done in the way of prevention.

From theſe obſervations it is evident,

That in certain parts of the world children are more ſubject to ſpaſmodic diſeaſes than others.

That theſe are more apt to come on within nine days after birth.

That coming on within this period they are generally productive of the moſt fatal effects.

And

And laftly,

That their caufes and cure are ever involved in obfcurity.

In each of thefe particulars, there is a ftriking analogy between the trifmus nafcentium or tetanus maxillæ inferioris and the nine-day fits.

It is farther worthy of obfervation, that the diforders of adults, which are confined to particular diftricts or tracts of country, more frequently arife from fomething noxious infecting the atmofphere of fuch places than from any other caufe; and however difficult it may be to apply this doctrine to the cafes in queftion, it at leaft affords fome probable evidence towards the fuppofition, that they originate from fomewhat fimilar caufes.

Such are the obfervations which reflection and fome reading fuggefted to me on this fubject, previous to the publication of the London Medical Tranfactions in the year 1785. In this very excellent work, however, I met with "An account of a fingular difeafe which pre-

" vailed among ſome poor children maintained " by the pariſh of St. James in Weſtminſter;" which appears to me to throw much light on this obſcure ſubject: I hope to be excuſed, therefore, for making ſome extracts from this valuable eſſay, for which the world is indebted to the accurate and learned Sir George Baker.

Sir George informs us, that on the 24th day of September, 1782, ſeventy-three children, viz. forty-ſix girls and twenty-ſeven boys, of different ages, from that of ſeven to fourteen years, were removed from Wimbleton to a large houſe near Golden-Square. To this houſe theſe children came in good health, and continued ſo for a fortnight; when on the 8th of October, a girl aged thirteen years was ſuddenly ſeized with an excruciating pain in the region of the ſtomach and in the back, which was ſoon followed by violent head-ach, delirium and convulſions. After a few days, another and another girl were attacked exactly in the ſame manner; and towards the end of the month this diſeaſe had ſo prevailed as very much to alarm all thoſe to whom the care of theſe children had been committed. On the 29th day of October Sir George's

George's advice was defired. He found nine of thefe poor girls and a female fervant in the fame room fuffering the various effects of a moft dreadful malady. Five were in the agonies of extreme pain, three were moft cruelly convulfed, and the other two were raving in a fit of delirium. The other inhabitants of this houfe had in general been healthy during the month of October, and it is remarkable that the difeafe above defcribed affected females only, and was confined to thofe who had flept together in a certain room on the fecond floor. The height of this room was a little more than eight feet, the length twenty, and the breadth fixteen: it contained ten beds, in which it was intended that eighteen girls, two in each bed, and a female fervant fingly fhould fleep; but Sir George difcovered that this being a favourite room on account of its warmth, was generally crouded at night by a much greater number than its complement: that as much fpace as poffible might be made for beds, the chimney had been ftopped up with bricks, and it had been the conftant cuftom of the fervant at night to keep the door fhut and to clofe the window fhutters, that as little frefh air as poffible might be admitted

On enquiry it appeared that three candles and a lamp of oil had been generally ufed during the night in this chamber, but they were hardly of any fervice, giving a glimmering light and frequently almoft extinguifhed.

Sir George advifed the chamber of the fick to be evacuated without delay, the healthy to be feparated from the difeafed, the chimney to be opened, and whatever tended to exclude frefh air to be removed. Thefe directions were complied with, and the patients having been removed to a large apartment (where proper care was taken that frefh air might be admitted) paffed a quiet night free from every fymptom of the difeafe. However, the next morning, immediately on their awaking, they were all feized in the ufual manner, but it was very foon obfervable, that the paroxyfms returned lefs often and with lefs violence, and fometimes without convulfions, and that during the intervals the delirium appeared gradually to abate.

From thefe and various other important facts which we cannot here recite, Sir George conjectures that the fource of this extraordinary difeafe was vitiated air. To me his evidence appears fufficient to afford conviction to every reafonable reaſonable mind, and if I am not miſtaken, it adds greatly to the probability of the opinion, which ſuppoſes that the nine-day fits originated from a ſimilar ſource.

Upon the whole, from the evidence adduced, I hope the following inferences may not appear improbable.

1. That one effect of an impure atmoſphere, on the human body, is to produce ſpaſms and convulſions.

2. That all young creatures, and eſpecially infants within nine days after birth, ſuffer moſt ſeverely by ſuch a noxious cauſe; and therefore

3. That in the conſtruction of lying-in hoſpitals, and perhaps of all public buildings intended for the reception of children, lofty ceilings, large windows and moderate ſized rooms ſhould be eſpecially attended to.

4. That in the arrangement of ſuch edifices, no apartment ſhould be completely filled with beds, if it can be conveniently avoided; and

5. That in their management attention is eſpecially neceſſary to cleanlineſs, as well as to the conſtant and uniform admiſſion of atmoſpheric air by night as well as by day; and

Laſtly. That by purſuing ſuch meaſures with care, diſeaſes may be prevented which it has hitherto been found difficult, and ſometimes impoſſible, to cure.

### An Abstract of the Registry * kept at the Lying-in Hospital, in Dublin,

From the 8th of December, 1757, (the Day it was first opened) to the 31st of December, 1788, each Year distinguished.

### By B. H. Register.

| | Number of Patients admitted. | Went out not delivered. | Delivered in the Hospital. | Boys born. | Girls born. | Total Number of Children. | Women having twins. | Children dead. | Children still-born. | Women dead. |
|---|---|---|---|---|---|---|---|---|---|---|
| From 8th to 31st December 1757 | 55 | — | 55 | 30 | 25 | 55 | — | 6 | 3 | 1 |
| Year ending 31st of December 1758 | 455 | 1 | 454 | 255 | 207 | 462 | 8 | 54 | 21 | 8 |
| 1759 | 413 | 7 | 406 | 228 | 192 | 420 | 13<br>1 had 3 | 95 | 22 | 5 |
| 1760 | 571 | 15 | 556 | 300 | 260 | 560 | 4 | 116 | 36 | 4 |
| 1761 | 537 | 16 | 521 | 283 | 249 | 532 | 11 | 104 | 29 | 9 |
| 1762 | 550 | 17 | 533 | 279 | 266 | 545 | 12 | 106 | 33 | 6 |
| 1763 | 519 | 31 | 488 | 274 | 224 | 498 | 12 | 94 | 29 | 9 |
| 1764 | 610 | 22 | 588 | 287 | 308 | 595 | 7 | 83 | 28 | 12 |
| 1765 | 559 | 26 | 533 | 288 | 251 | 539 | 6 | 94 | 25 | 6 |
| 1766 | 611 | 30 | 581 | 324 | 261 | 585 | 4 | 111 | 18 | 3 |
| 1767 | 695 | 31 | 664 | 373 | 301 | 674 | 10 | 125 | 29 | 11 |
| 1768 | 689 | 34 | 655 | 362 | 302 | 664 | 9 | 154 | 47 | 16 |
| 1769 | 675 | 33 | 642 | 350 | 301 | 651 | 9 | 152 | 38 | 8 |
| 1770 | 705 | 35 | 670 | 372 | 305 | 677 | 7 | 107 | 37 | 8 |
| 1771 | 724 | 29 | 695 | 370 | 341 | 711 | 16 | 102 | 44 | 5 |
| 1772 | 725 | 21 | 704 | 368 | 344 | 712 | 8 | 116 | 32 | 4 |
| 1773 | 727 | 33 | 694 | 367 | 344 | 711 | 17 | 136 | 31 | 13 |
| 1774 | 709 | 28 | 681 | 357 | 334 | 691 | 10 | 154 | 29 | 21 |
| 1775 | 752 | 24 | 728 | 364 | 378 | 742 | 14 | 122 | 27 | 5 |
| 1776 | 883 | 31 | 802 | 418 | 407 | 825 | 22<br>1 had 3 | 132 | 39 | 7 |
| 1777 | 872 | 37 | 835 | 452 | 395 | 847 | 12 | 145 | 35 | 7 |
| 1778 | 961 | 34 | 927 | 476 | 460 | 936 | 9 | 127 | 39 | 10 |
| 1779 | 1064 | 53 | 1011 | 550 | 476 | 1026 | 15 | 146 | 59 | 8 |
| 1780 | 967 | 48 | 919 | 499 | 441 | 940 | 21 | 115 | 41 | 5 |
| 1781 | 1079 | 52 | 1027 | 598 | 447 | 1045 | 18 | 121 | 38 | 6 |
| 1782 | 1021 | 31 | 990 | 549 | 458 | 1007 | 17 | 127 | 57 | 6 |
| 1783 | 1230 | 63 | 1167 | 632 | 553 | 1185 | 17<br>1 had 3 | 91 | 72 | 15 |
| 1784 | 1317 | 56 | 1261 | 643 | 641 | 1284 | 24 | 76 | 68 | 11 |
| 1785 | 1349 | 57 | 1292 | 711 | 609 | 1320 | 28<br>1 had 3 | 87 | 75 | 8 |
| 1786 | 1396 | 45 | 1351 | 716 | 656 | 1372 | 21 | 51 | 101 | 8 |
| 1787 | 1418 | 71 | 1347 | 705 | 670 | 1375 | 28 | 59 | 95 | 14 |
| 1788 | 1533 | 64 | 1469 | 725 | 771 | 1496 | 25<br>1 had 4 | 55 | 72 | 23 |
| Totals | 26321 | 1075 | 25246 | 13505 | 12177 | 25682 | 432 | 3363 | 1349 | 282 |

Proportion of males and females born, about *nine* males to *eight* females.
——— children dying in the hospital, as *one* to about *seven*.
——— children still born, as *one* to about *nineteen*.
——— women having twins (and more), as *one* to about *fifty eight*.
——— women dying in childbed, as *one* to about *ninety*.
——— women having *three* (and *four*) children, as *one* to about *five thousand and fifty*.

* An abstract of this Registry, from 1757 to 1784, was annexed by Dr. Clarke to his letters to the late Dr. Price, (see Philosophical Transactions, Vol. LXXVI. and London Medical Journal, Vol. IX.); *but the present abstract, being brought down to a later period, includes a much greater number of facts, and, in particular, shows the decreased mortality of children in the Hospital since the year 1783.*—Editor.

